# Quality of life after pediatric cancer: comparison of long-term childhood cancer survivors’ quality of life with a representative general population sample and associations with physical health and risk indicators

**DOI:** 10.1186/s12955-023-02153-7

**Published:** 2023-07-04

**Authors:** Mareike Ernst, Andreas Hinz, Elmar Brähler, Hiltrud Merzenich, Jörg Faber, Philipp S. Wild, Manfred E. Beutel

**Affiliations:** 1 Department of Psychosomatic Medicine and Psychotherapy, University Medical Center of the Johannes Gutenberg-University Mainz, Mainz, Germany; 2 Department of Clinical Psychology, Psychotherapy and Psychoanalysis, Institute of Psychology, University of Klagenfurt, Klagenfurt Am Wörthersee, Austria; 3 Department of Medical Psychology and Medical Sociology, University of Leipzig, Leipzig, Germany; 4 Department of Psychosomatic Medicine and Psychotherapy, Integrated Research and Treatment Center Adiposity Diseases, University of Leipzig Medical Center, Leipzig, Germany; 5 Institute for Medical Biostatistics, Epidemiology and Informatics, University Medical Center of the Johannes Gutenberg-University Mainz, Mainz, Germany; 6 Department of Pediatric Hematology/Oncology/Hemostaseology, Center for Pediatric and Adolescent Medicine, University Medical Center of the Johannes Gutenberg-University Mainz, Mainz, Germany; 7 Preventive Cardiology and Preventive Medicine, Department of Cardiology, University Medical Center of the Johannes Gutenberg-University Mainz, Mainz, Germany; 8 Clinical Epidemiology and Systems Medicine, Center for Thrombosis and Hemostasis (CTH), University Medical Center of the Johannes Gutenberg-University Mainz, Mainz, Germany; 9 German Center for Cardiovascular Research (DZHK), Partner Site Rhine-Main, Mainz, Germany; 10 Institute of Molecular Biology (IMB), Mainz, Germany

**Keywords:** Cancer survivorship, Childhood cancer, Long-term survival, Quality of life, Risk factors, Risk indicators

## Abstract

**Background:**

This study aimed to compare the quality of life (QoL) reported by childhood cancer survivors (CCS) drawn from a cohort of the German Childhood Cancer Registry with a representative general population sample and, within CCS, to test associations between QoL and health behavior, health risk factors, and physical illness.

**Methods:**

CCS (*N* = 633, age at diagnosis *M* = 6.34 (*SD* = 4.38), age at medical assessment *M* = 34.92 (*SD* = 5.70)) and a general population sample (age-aligned; *N* = 975) filled out the EORTC QLQ-C30. Comparisons were performed using General linear models (GLMs) (fixed effects: sex/gender, group (CCS vs. general population); covariates: age, education level). CCS underwent an extensive medical assessment (mean time from diagnosis to assessment was 28.07 (*SD* = 3.21) years) including an objective diagnosis of health risk factors and physical illnesses (e.g., diabetes and cardiovascular disease). Within CCS, we tested associations between QoL and sociodemographic characteristics, health behavior, health risk factors, and physical illness.

**Results:**

CCS, especially female CCS, reported both worse functional QoL and higher symptom burden than the general population. Among CCS, better total QoL was related to younger age, higher level of education, being married, and engaging in active sports. Both health risk factors (dyslipidemia and physical inactivity) and manifest physical illnesses (cardiovascular disease) were associated with lower total QoL.

**Conclusions:**

In all domains, long-term CCS reported worse QoL than the comparison sample. The negative associations with risk factors and physical illnesses indicate an urgent need for long-term surveillance and health promotion.

**Supplementary Information:**

The online version contains supplementary material available at 10.1186/s12955-023-02153-7.

## Background

Following great medical advances in the treatment of childhood cancer, long-term survival rates have for years surpassed 80% and the quality of life (QoL) of former patients has become a (psycho-)oncological research focus. Especially as childhood cancer survivors (CCS) reach middle and late adulthood, their risk for physical late effects, which can significantly diminish well-being, increases [1–3]. Previous research has identified numerous risk indicators for poor QoL outcomes in CCS including disease- and treatment-related variables (such as a CNS tumor diagnosis, see [4–6] for reviews). Furthermore, as childhood cancer has implications for all areas of life including the social and psychological domain [7], researchers have noted the importance of studying survivors’ physical health in relation to other factors such as psychosocial adjustment [4, 8]. Psychosocial characteristics had relevant associations with QoL outcomes in numerous studies [2, 4, 5]: In a large sample of adult CCS drawn from a Dutch cohort (surveyed with the SF-36), single status and low educational attainment were associated with poorer outcomes in both domains. Women were particularly at risk for poor physical QoL and men for poor mental QoL [4]. By contrast, previous comparisons of the general population with young adolescent cancer survivors in Germany reported that female survivors reported the worst quality of life in all domains of the EORTC QLQ-C30 [9]. In this sample, QoL did not differ according to relationship status.

However, there are only a few large-scale empirical investigations of CCS using comprehensive QoL instruments that are in line with the biopsychosocial model [8] in the sense that they include medical assessments and information about modifiers of mental and physical health from other domains of life (such as educational attainment, relationship status, and health behavior) [5]. Such studies would yield valuable information to stratify prevention intervention efforts in the both growing and aging CCS population. To fill this research gap, this study provides both a comparison of long-term CCS’ responses to the EORTC QLQ-C30 with those of a representative general population sample and an investigation of associations of QoL with sociodemographic and psychosocial factors and health risk factors and physical illnesses representing common late effects. This was done in a sex/gender-sensitive way by not just comparing men and women, but also considering the interaction of sex/gender with long-term cancer survival.

## Material and Methods

### Participants and procedure

#### Childhood cancer survivors

**Fig. 1:**
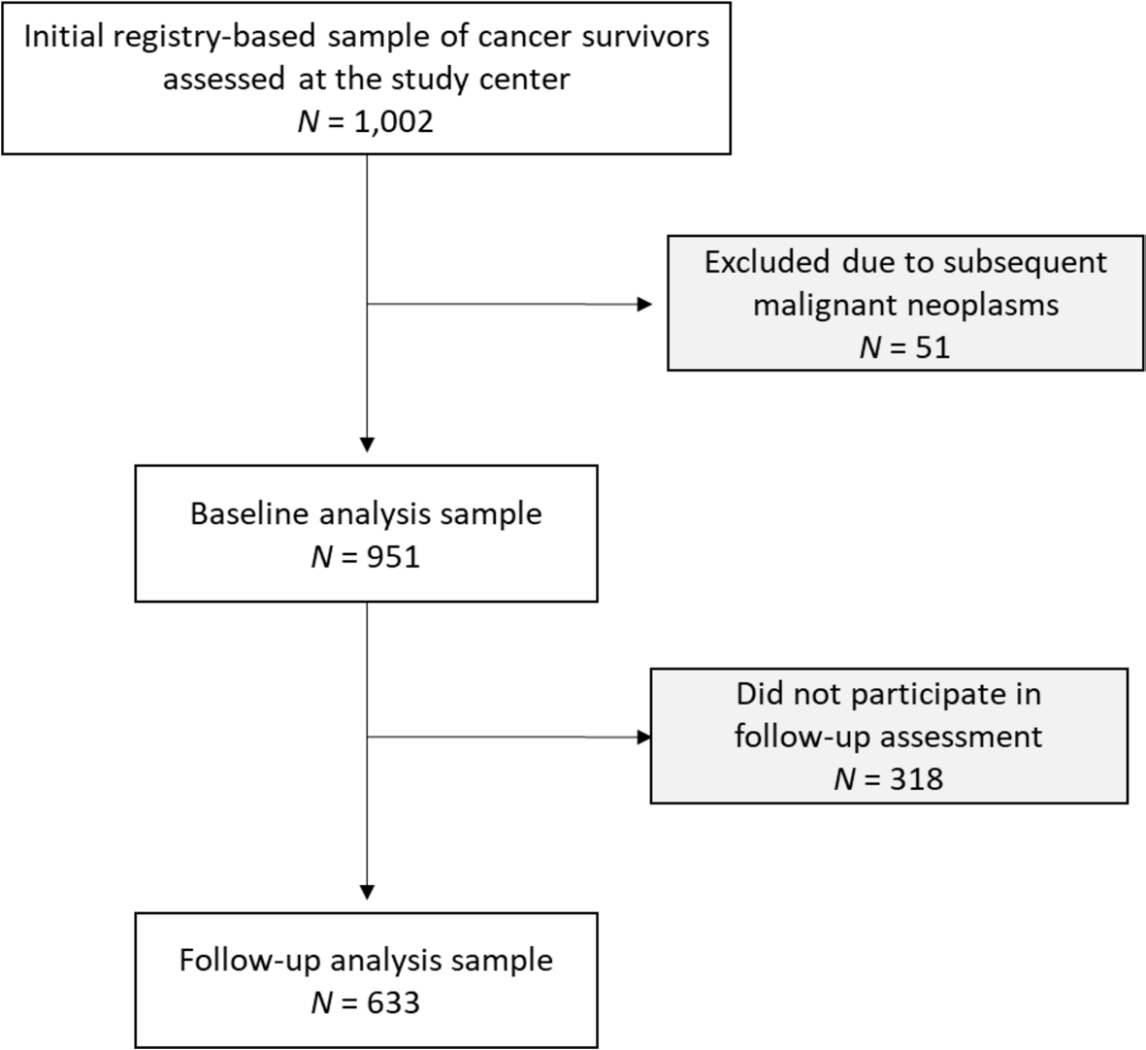
Participant flow for the childhood cancer survivor sample

CCS were recruited in cooperation with the German Childhood Cancer Registry (GCCR). The nationwide GCCR systematically documents patients with childhood cancer residing in Germany since 1980 [10]. German CCS were eligible for participation if diagnosed with neoplasia according to the International Classification of Childhood Cancer (ICCC-3) [11] between 1980 and 1990 before the age of 15, if registered at the GCCR, and if they had received antineoplastic treatment at one of 34 participating pediatric cancer centers. Survivors of Hodgkin lymphoma and a small group of former nephroblastoma patients could not be enrolled as they had taken part in other trials. A total of 2,894 eligible survivors were invited to take part in the studies CVSS (Cardiac and Vascular late Sequelae in long-term Survivors of Childhood Cancer, clinicaltrials.gov-nr.: NCT02181049) and PSYNA (Psychosocial long-term effects, health behavior, and prevention among long-term survivors of cancer in childhood and adolescence). This invitation was accepted by 1,002 CCS who were medically examined at the study center (between 2013/09 and 2016/02). After excluding 51 individuals due to subsequent malignant neoplasms, the baseline sample included 951 participants. A second assessment 1.5–2 years later consisted of a computer-assisted personal interview (CAPI) on health status and medical history, and mailed questionnaires concerning psychosocial aspects which included the EORTC QLQ-C30. As part of this second assessment, the EORTC QLQ-C30 was completed by 633 (44.4% women) individuals who constitute the sample of this investigation (see Fig. 1). The study procedure, participants’ diagnoses, and treatment-related information are described in more detail elsewhere [12].

CVSS and PSYNA are carried out in accordance with the ethical standards of the institutional research committee (approved by the ethics review committee of Rhineland-Palatinate Chamber of Physicians, nr. 837.453.13(9138-F)) and with the Declaration of Helsinki. Participants gave written informed consent for study participation and data retrieval.

#### Representative general population sample

In cooperation with the independent demography research institute USUMA Berlin, data of a representative general population sample were collected in 2012. After predefining 258 German regional areas using the reference system for representative studies in Germany provided by the ADM-Sampling-System, target households within these regional areas were selected following a random route procedure. For multi-person households, one person was randomly selected using the Kish grid [13]. To be eligible for survey inclusion, participants had to be at least 14 years of age and have sufficient German language skills. Anonymity in responses was guaranteed and all respondents provided informed consent before taking part in the study. The survey procedure and materials were approved by the ethics committee of the Medical Faculty of the University of Leipzig (nr. 092‐12‐05032012). For this work, we aligned the representative sample’s age to the CCS sample by excluding younger (< 23 years) and older (> 48 years) participants. This reduced the general population sample from 2,510 to 975 participants (53.7% women).

#### Data and measures

*Sociodemographic information* including date of birth, sex/gender, level of education (with high education denoting the German Abitur/equivalent qualification ranked 4 in the European Qualifications Framework (EQF), required for postsecondary education at universities), marital status, parenthood, and level of education was assessed via self-report as part of the CAPI at the study center in the case of CCS. In the general population sample, face-to-face interviews were conducted at participants’ homes.

CCS’ *illness- and treatment-related information* was abstracted from primary health records of former treating medical centers and/or centrally documented individual therapy data available at the Society for Pediatric Oncology and Hematology’s (GPOH) study centers. It was validated by trained medical staff.

*Health behavior* in CCS included smoking, active sports, and alcohol consumption.

Participants’ reports were dichotomized into nonsmokers and smokers (the latter combining occasional and frequent smokers).

The SQUASH ("Short QUestionnaire to ASsess Health enhancing physical activity") [14] captures commuting, leisure time, household, work, and school activities with reference to a typical week in recent months. Sleeping, lying, sitting, and standing were classified as inactivity. Physical activity was presented in quartiles with Q1 denominating the lowest and Q4 the highest quartile of physical activity. The highest quartile of physical activity was coded as active sports.

Alcohol consumption was assessed via self-report. Participants reported how often, how many, and which kinds of beverages they consumed (e.g., beer, wine, spirits). Following a standardized procedure, the total amount (in grams/day) was calculated from these responses. Alcohol consumption surpassing the recommended limits was defined in line with the German threshold for alcohol consumption above tolerance (≥ 10 g/day in women; ≥ 20 g/day in men).

*Health risk factors and physical illnesses* were diagnosed in CCS based on highly standardized, 5.5-h medical examinations conducted at the study center which used the platform of the Gutenberg Health Study (GHS) (details in [12, 15]). It included the following illnesses and health risk factors: cardiovascular disease (CVD), diabetes, arterial hypertension, dyslipidemia, chronic obstructive pulmonary disease (COPD), chronic kidney disease, chronic liver disease, and obesity. Information was collected through a computer-assisted personal interview (CAPI), from medical records and medication packages, and, if applicable, using clinical and laboratory examinations. CVD was defined when diagnosed by a physician and ascertained by medical records; it included congestive heart failure (CHF) (requiring medication in the last 12 months), coronary heart disease including myocardial infarction, stroke, peripheral artery disease, atrial fibrillation, and venous thromboembolism (VTE) including deep venous thrombosis and pulmonary embolism. Obesity was defined as a Body Mass Index (BMI) of at least 30 kg/m^2^). Diabetes was defined in individuals with a diagnosis of diabetes by a physician and/or intake of antidiabetic medication within the past two weeks and/or HBA1c ≥ 6.5% (Glycated hemoglobin A1c). Dyslipidemia was also defined as a physician’s diagnosis and/or LDL/HDL ratio > 3.5 (low-density lipoprotein/high-density lipoprotein) and/or a fasting blood triglyceride level ≥ 150 mg/dl.

*Quality of life* was assessed using the EORTC QLQ-C30 [16] in both samples. It comprises 30 items (five functioning scales: physical, role, emotional, social, cognitive; three symptom scales: fatigue, pain, nausea/vomiting; global health status/QoL scale comprising two items; single items dyspnea, appetite loss, insomnia, constipation, diarrhea, financial difficulties). In line with the manual [17], scales and single items were transformed to range from 0 to 100.

The sum score was calculated following Giesinger, Kieffer [18, 19] as the mean of the five functioning scales and the symptom scales (excluding global QoL and financial difficulties).

#### Statistical procedure

The interpretation of regression coefficients and effect sizes (partial *η*^*2*^, Cohen’s *d*) follows Cohen [20]. Analyses were conducted using R version 4.0.3. *P*-values denote two-tailed tests, with *p* < 0.05 being considered statistically significant. Due to the exploratory nature of the present investigation, we made no adjustments for multiple comparisons. However, we report the sizes of observed effects so that readers can better interpret their relative magnitude and relevance.

Comparisons were carried out as General Linear Models (GLMs) (fixed effects: sample (general population vs. CCS), sex/gender (men vs. women); covariates: age (continuous), level of education (low vs. high).

Within CCS, we calculated associations of sociodemographic and health behavior variables as independent t-tests and correlation analyses. The relevance of health risk factors and physical illnesses for QoL was tested in a linear regression model which also included the covariates age, gender, and level of education.

## Results

### Sample characteristics

The CCS sample represented 22% of the eligible total cohort of German CCS and comprised 352 men and 281 women. The general population sample comprised 451 men and 524 women. Among CCS, the largest diagnosis group was leukemias (42.2%, *n* = 267). Their mean age at diagnosis was 6.34 years (*SD* = 4.38) and the mean follow-up time since then was 28.07 (*SD* = 3.21) years (Table 1).

**Table 1:** Sample characteristics including mean values and standard deviations on the EORTC QLQ-C30 subscales

	Childhood cancer survivors						General population sample					
	All *N* = 633		Men *N* = 352		Women *N* = 281		All *N* = 975		Men *N* = 451		Women *N* = 524	
Sociodemographic characteristics												
Age at study assessment (*M*, *SD*)	34.92	5.70	35.42	5.63	34.29	5.73	36.71	7.75	36.47	7.80	36.93	7.71
High level of education (*N*, %)	273	43.12	157	44.60	116	41.3	207	21.2	109	24.17	98	18.70
Married (*N*, %)	233	36.9	131	37.3	102	36.3	501	51.4	223	49.45	278	53.05
Age at diagnosis (*M*, *SD*)	6.34	4.38	6.69	4.44	5.90	4.29	-	-	-	-	-	-
Years since diagnosis (*M*, *SD*)	28.07	3.21	28.22	3.10	27.88	3.33	-	-	-	-	-	-
EORTC QLQ-C30 (*M*, *SD*)												
Total QoL (sum score)	86.26	14.25	89.25	12.20	82.52	15.69	94.40	10.13	95.26	9.13	93.66	10.87
Global QoL (2 items)	74.02	20.30	77.43	18.46	69.77	21.67	81.27	17.36	82.19	17.19	80.48	17.48
Functional scales												
*Physical function (PF)*	92.35	14.63	94.99	11.85	89.05	16.93	96.96	10.01	97.60	9.07	96.41	10.74
*Role function (RF)*	86.55	23.06	89.51	21.42	82.86	24.51	94.55	16.57	95.40	16.36	93.83	16.72
*Emotional function (EF)*	74.19	24.51	78.78	21.99	68.45	26.27	84.73	19.56	85.94	19.84	83.69	19.27
*Cognitive function (CF)*	84.02	22.80	87.04	19.53	80.25	25.87	95.77	12.49	96.40	10.97	95.22	13.65
*Social function (SF)*	85.10	24.38	87.81	22.37	81.73	26.32	95.23	15.02	96.04	14.58	94.54	15.37
Symptom scales												
*Fatigue*	25.39	23.93	20.45	21.75	31.55	25.11	11.27	18.89	9.23	17.59	13.02	19.79
*Nausea/vomiting*	3.49	10.51	2.33	9.27	4.94	11.74	1.60	7.64	0.89	5.03	2.20	9.28
*Pain*	18.96	26.23	13.96	23.09	25.21	28.53	9.89	20.61	9.09	20.73	10.58	20.49
*Dyspnea*^a^	9.28	20.43	6.93	17.62	12.22	23.17	3.08	12.54	2.07	10.45	3.95	14.04
*Insomnia*^a^	22.47	29.82	18.14	27.08	27.88	32.15	8.17	20.16	7.83	20.43	8.46	19.94
*Appetite loss*^a^	4.38	13.80	3.13	10.68	5.93	16.80	2.87	12.58	1.77	10.51	3.82	14.07
*Constipation*^a^	6.43	18.60	2.94	11.58	10.79	24.03	1.37	7.88	0.67	5.63	1.98	9.36
*Diarrhea*^a^	9.65	20.39	9.40	19.44	9.96	21.54	2.46	12.21	0.96	6.41	3.75	15.45
*Financial difficulties*^a^	8.15	22.65	5.43	17.27	11.55	27.61	3.59	14.97	3.34	15.47	3.80	14.53

^a^Indicates single items. QoL = quality of life. High level of education denotes the German Abitur which qualifies for university admission (usually obtained after 12–13 years of school)

### QoL comparisons

**Fig. 2:**
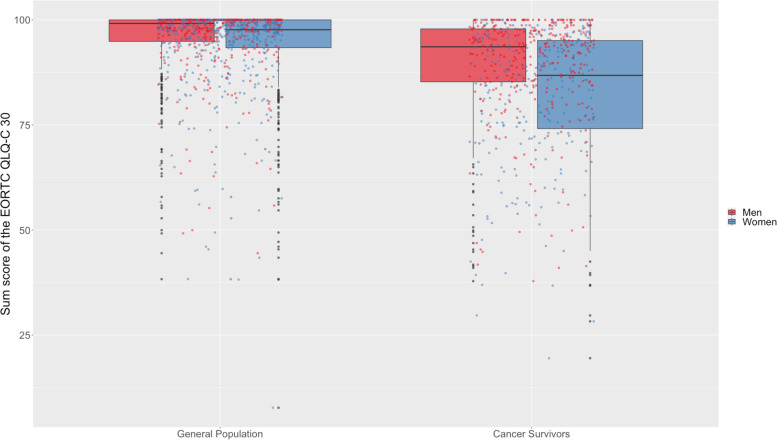
Plot of participants’ quality of life reports Interaction between sex/gender and group (general population vs. long-term childhood cancer survivors). The sum score of the EORTC QLQ-C30 includes the functional and symptom scales (excluding financial difficulties), with higher scores indicating better quality of life (range 0–100)

Regarding the sum score (Table 2), there were moderate group differences: CCS reported worse functioning/more symptoms than the comparison sample. We also observed small differences between men and women with worse QoL in women, and a small interaction effect of sex/gender and group (i.e., female CCS reported particularly bad outcomes) which is visualized in Fig. 2. Supplementary Fig. 1 yields a separate depiction of the different groups' values on the five functioning scales and the three symptom scales.

**Table 2:** Results of the General linear models comparing quality of life (QoL) in cancer survivors and the general population

	*F*	*p*	*η*^*2*^
Total QoL (sum score) (adj. *R*^2^ = .142)			
Group	203.969	** < .001**	.115
Sex/gender	46.808	** < .001**	.029
Sex/gender x Group	19.664	** < .001**	.012
Age	12.045	** < .001**	.008
Level of education	5.916	**.015**	.004

Statistically significant effects are printed in bold

The Suppl. Tables depict the results of the GLMs of the single EORTC QLQ-C30 subscales and items. The models of the sum score, cognitive functioning, and fatigue explained the largest proportions of variance. These models also included the largest group differences, however, comparatively worse outcomes in women and worse outcomes in CCS were observed throughout. Interaction effects, in the direction that female CCS reported particularly bad outcomes, were present in most models (exceptions: nausea/vomiting, appetite loss, and diarrhea).

### Associations of QoL with sociodemographic characteristics, health behavior, and physical health within CCS

There were no statistically significant differences between the four main diagnosis groups concerning total QoL (leukemias: *n* = 267, *M* = 86.42, *SD* = 14.5; lymphomas: *n* = 64, *M* = 88.36, *SD* = 13.08; CNS tumors: *n* = 84,* M* = 84.49, *SD* = 16.08; others: *n* = 218, *M* = 85.59, *SD* = 13.94).

Higher age was related to lower total QoL (*r* = -0.098, *p* = 0.014), but there was no statistically significant association with time since diagnosis. CCS with higher educational attainment reported better total QoL (*d* = 0.34, *p* < 0.001). Total QoL was higher in married CCS (*d* = 0.20, *p* = 0.016), but there were no statistically significant differences between CCS with and without children. CCS engaging in active sports reported higher total QoL (*d* = 0.32, *p* < 0.001). There was no statistically significant association of QoL with smoking or alcohol consumption.

In the linear regression analysis (adj. R^2^ = 0.129), health risk factors and physical illnesses were independently associated with lower total QoL (Table 3): statistically significant predictors were the presence of cardiovascular disease, physical inactivity, and dyslipidemia.

**Table 3:** Linear regression model of total QoL on health risk behavior and physical illnesses within the cancer survivor sample

	*B* (*SE*)	*β*	*p*
Intercept	103.015 (4.122)		** < .001**
Sex/gender	-6.639 (1.170)	-.229	** < .001**
Age at examination (continuous)	-.178 (.103)	-.071	.085
High level of education	3.537 (1.164)	.122	**.002**
Smoking status	.649 (1.214)	.022	.593
Alcohol consumption above tolerance	.687 (1.871)	.015	.714
Physical inactivity	-2.840 (1.238)	-.093	**.022**
Obesity	-1.370 (1.604)	-.035	.393
Dyslipidemia	-2.680 (1.317)	-.084	**.042**
Hypertension	.113 (1.426)	.003	.937
Chronic kidney disease	-2.240 (4.743)	-.019	.637
Chronic obstructive pulmonary disease	-4.077 (3.924)	-.042	.299
Cardiovascular disease	-12.331 (2.973)	-.168	** < .001**
Diabetes	-6.162 (4.336)	-.059	.156

Statistically significant effects are printed in bold. Coding of binary predictors: Gender: 0 = men, 1 = women; Level of education: 0 = below German Abitur; 1 = German Abitur or higher; Smoking status; Alcohol consumption above tolerance; Physical inactivity; Obesity; Dyslipidemia; Hypertension; Chronic kidney disease; Chronic obstructive pulmonary disease; Cardiovascular disease; Diabetes: 0 = not present, 1 = present

## Discussion

In the present investigation, German CCS reported worse QoL than a representative general population sample, mirroring previous registry-based Italian [21] and Swiss [22] studies and an investigation from the Netherlands conducted through follow-up clinics [2], all of which had also included comparison groups (drawn from the community or CCS’ siblings). The present study suggests diminished QoL in all areas surveyed. Together with the negative associations of QoL with single status and lower educational attainment which are in line with previous findings [5, 6, 23], this indicates survivors’ need for multi-professional survivorship care that also addresses the psychological and social domain [7, 8].

While previous research had found worse health-related QoL and more severe late effects in survivors with longer follow-up times [2], within our sample, we observed effects of age rather than associations with follow-up time. Women have also been noted as a risk group for worse health-related QoL outcomes after childhood cancer [2, 9, 21, 23], including a report of German and Austrian long-term survivors of Hodgkin’s lymphoma [24], underscoring the relevance of considering CCS’ sex/gender as a modifying factor. Although underlying mechanisms are not clear, worse outcomes in female CCS, especially mental health, have often been observed [7, 25], yielding important clues for risk stratification of screening and follow-up care.

We did not find associations of all kinds of health risk behavior with participants’ QoL, perhaps indicating that the negative effects of some behaviors might not yet become apparent in early/middle adulthood. However, given CCS’ health vulnerabilities [3] (as indicated by e.g., early onset of CVD and cardiovascular risk factors [12]), health behavior is an important target for efforts to foster CCS’ long-term QoL. The negative effects of dyslipidemia and physical inactivity on QoL observed in this study suggest that modifiable physical health risk factors, not just manifest diseases, have an impact on CCS’ QoL. These findings expand previous investigations which had investigated associations with chronic conditions [2].

### Strengths and limitations

Although this study relied on a registry-based sample of CCS, it did not include the whole target cohort. The response rate of 33% among invited CCS cautions against generalizations. Self-selection effects cannot be ruled out, especially as participation implied traveling to the study center and taking part in extensive medical assessments. The exclusion of CCS with secondary neoplasia limits the comparability with other studies that included CCS with secondary neoplasia and the present results’ generalizability to the entirety of CCS.

The highly standardized medical assessment is an asset of the present study. However, there was too little information about CCS’ past cancer treatment to test more specific consequences for survivors’ QoL, e.g., of a stem cell transplant, chemotherapeutic agents, radiation dose (factors that previously influenced late effects [5]). While we assessed several health risk factors and physical illnesses, we could not address all potential late effects that are relevant for CCS’ QoL (such as hearing loss, osteoporosis, infertility, etc. [3]). The cross-sectional study design does not allow to test the directions of observed associations (e.g., of physical activity and QoL). However, this work presents the most comprehensive investigation of German long-term CCS’ QoL and influencing factors to date, highlighting the need to address healthcare disparities to ensure better health for all CCS [26].

## Conclusion

Long-term CCS reported worse QoL than the general population. Expanding on previous research, health risk factors and physical illnesses were associated with lower QoL, indicating promising targets for prevention and intervention efforts. The present findings underscore the need for long-term surveillance and have implications for risk stratification as risk indicators also include sociodemographic and psychosocial factors, highlighting that CCS’ well-being needs to be seen in the context of the rest of their life which includes resources and late effects in other areas of life.

## Supplementary Information

**Additional file 1:** **Supplementary Tables.** Results of the general linear models for all EORTC QLQ-C30 subscales and single items. **Supplementary Figure 1.** Interaction between sex/gender and group. Sum scores of the five functional and three symptom scales, with higher scores indicating better quality of life and more symptoms, respectively.

## Data Availability

The written informed consent of the study participants is not suitable for public access to the data and this concept was not approved by the local data protection officer and ethics committee. Access to data at the local database in accordance with the ethics vote is offered upon request at any time. Interested researchers make their requests to the Principal Investigators of the CVSS/PSYNA study (Philipp.Wild@unimedizin-mainz.de).
