# Supplementary material for: Quality of life after pediatric cancer: comparison of long-term childhood cancer survivors’ quality of life with a representative general population sample and associations with physical health and risk indicators
Source: Health Qual Life Outcomes. 2023 Jul 4;21:65. doi: 10.1186/s12955-023-02153-7 (PMC10318771; doi:10.1186/s12955-023-02153-7)
Supplement: Supplementary file 1 — Additional file 1: Supplementary Tables. Results of the general linear models for all EORTC QLQ-C30 subscales and single items. Supplementary Figure 1. Interaction between sex/gender and group. Sum scores of the five functional and three symptom scales, with higher scores indicating better quality of life and more symptoms, respectively. [file 12955_2023_2153_MOESM1_ESM.docx]

**Supplementary Tables**

Results of the general linear models for all EORTC QLQ-C30 subscales and single items

I Functional scales

|  | *F* | *p* | *η^2^* |
| --- | --- | --- | --- |
| Physical function (PF) (adj. R^2^ = .080) |  |  |  |
| Group | 78.378 | **< .001** | .047 |
| Sex/gender | 33.624 | **< .001** | .021 |
| Sex/gender x Group | 17.864 | **< .001** | .011 |
| Age | 23.957 | **< .001** | .015 |
| Level of education | 15.052 | **< .001** | .009 |
| Role function (RF) (adj. R^2^ = .060) |  |  |  |
| Group | 80.643 | **< .001** | .048 |
| Sex/gender | 16.686 | **< .001** | .010 |
| Sex/gender x Group | 7.747 | **.005** | .005 |
| Age | 9.566 | **.002** | .006 |
| Level of education | 8.767 | **.003** | .005 |
| Emotional function (EF) (adj. R^2^ = .077) |  |  |  |
| Group | 99.830 | **< .001** | .059 |
| Sex/gender | 33.057 | **< .001** | .020 |
| Sex/gender x Group | 14.395 | **< .001** | .009 |
| Age | 5.899 | **.015** | .004 |
| Level of education | 0.201 | .654 | .000 |
| Cognitive function (CF) (adj. R^2^ = .122) |  |  |  |
| Group | 192.999 | **< .001** | .108 |
| Sex/gender | 20.530 | **< .001** | .013 |
| Sex/gender x Group | 11.692 | **.001** | .007 |
| Age | 15.003 | **< .001** | .009 |
| Level of education | 3.814 | .051 | .002 |
| Social function (SF) (adj. R^2^ = .078) |  |  |  |
| Group | 117.649 | **< .001** | .069 |
| Sex/gender | 14.728 | **< .001** | .009 |
| Sex/gender x Group | 6.446 | **.011** | .004 |
| Age | 10.747 | **.001** | .007 |
| Level of education | 3.704 | .054 | .002 |

*Note.* Statistically significant effects are printed in bold.

II Symptom scales/items

|  | *F* | *p* | *η^2^* |
| --- | --- | --- | --- |
| Fatigue (adj. R^2^ = .128) |  |  |  |
| Group | 188.945 | **< .001** | .106 |
| Sex/gender | 49.611 | **< .001** | .030 |
| Sex/gender x Group | 12.926 | **< .001** | .008 |
| Age | 6.889 | **.009** | .004 |
| Level of education | 0.343 | .558 | .000 |
| Nausea/vomiting (adj. R^2^ = .021) |  |  |  |
| Group | 23.505 | **< .001** | .015 |
| Sex/gender | 17.751 | **< .001** | .011 |
| Sex/gender x Group | 1.965 | .161 | .001 |
| Age | 0.977 | .323 | .001 |
| Level of education | 2.463 | .117 | .002 |
| Pain (adj. R^2^ = .074) |  |  |  |
| Group | 77.383 | **< .001** | .046 |
| Sex/gender | 29.786 | **< .001** | .018 |
| Sex/gender x Group | 20.338 | **< .001** | .013 |
| Age | 21.389 | **< .001** | .013 |
| Level of education | 7.866 | **.005** | .005 |
| Dyspnea (adj. R^2^ = .048) |  |  |  |
| Group | 65.167 | **< .001** | .039 |
| Sex/gender | 18.947 | **< .001** | .012 |
| Sex/gender x Group | 4.925 | **.027** | .003 |
| Age | 6.025 | **.014** | .004 |
| Level of education | 1.884 | .170 | .001 |
| Insomnia (adj. R^2^ = .093) |  |  |  |
| Group | 139.428 | **< .001** | .080 |
| Sex/gender | 17.605 | **< .001** | .011 |
| Sex/gender x Group | 14.698 | **< .001** | .009 |
| Age | 8.415 | **.004** | .005 |
| Level of education | 0.683 | .409 | .000 |
| Appetite loss (adj. R^2^ = .008) |  |  |  |
| Group | 6.734 | **.010** | .004 |
| Sex/gender | 13.003 | **< .001** | .008 |
| Sex/gender x Group | 0.345 | .557 | .000 |
| Age | 0.145 | .704 | .000 |
| Level of education | 0.099 | .753 | .000 |

|  | *F* | *p* | *η^2^* |
| --- | --- | --- | --- |
| Constipation (adj. R^2^ = .068) |  |  |  |
| Group | 72.933 | **< .001** | .044 |
| Sex/gender | 46.166 | **< .001** | .028 |
| Sex/gender x Group | 24.293 | **< .001** | .015 |
| Age | 0.009 | .926 | .000 |
| Level of education | 3.228 | .073 | .002 |
| Diarrhea (adj. R^2^ = .051) |  |  |  |
| Group | 86.238 | **< .001** | .051 |
| Sex/gender | 3.760 | .053 | .002 |
| Sex/gender x Group | 1.793 | .181 | .001 |
| Age | 0.000 | .977 | .000 |
| Level of education | 5.583 | **.018** | .003 |
| Financial difficulties (adj. R^2^ = .040) |  |  |  |
| Group | 34.399 | **< .001** | .021 |
| Sex/gender | 11.831 | **.001** | .007 |
| Sex/gender x Group | 10.779 | **.001** | .007 |
| Age | 15.255 | **< .001** | .009 |
| Level of education | 11.572 | **.001** | .007 |

*Note.* Statistically significant effects are printed in bold.

III Global QoL (two items)

|  | *F* | *p* | *η^2^* |
| --- | --- | --- | --- |
| Global QoL (adj. R^2^ = .072) |  |  |  |
| Group | 79.160 | **< .001** | .047 |
| Sex/gender | 24.095 | **< .001** | .015 |
| Sex/gender x Group | 12.047 | **.001** | .007 |
| Age | 20.160 | **< .001** | .012 |
| Level of education | 14.884 | **< .001** | .009 |

*Note.* Statistically significant effects are printed in bold.

**Supplementary Figure 1**

**
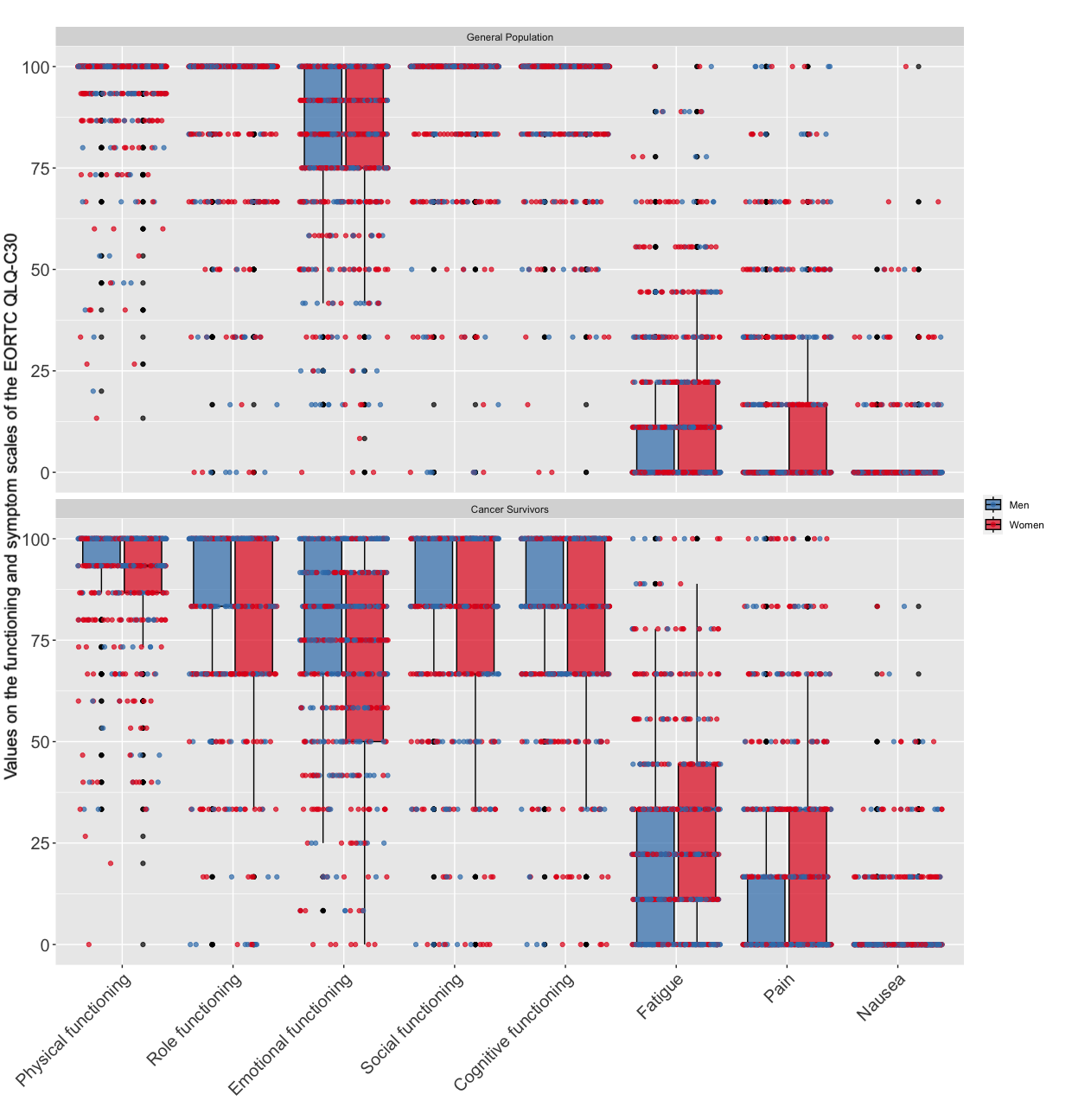
**

Figure legend. Plot of participants’ quality of life reports

Interaction between sex/gender and group (general population at the top and long-term childhood cancer survivors at the bottom). Sum scores of the five functional and three symptom scales, with higher scores indicating better quality of life and more symptoms, respectively.
